# Phosphorus availability and planting patterns regulate soil microbial effects on plant performance in a semiarid steppe

**DOI:** 10.1093/aob/mcad012

**Published:** 2023-01-20

**Authors:** Yawen Li, Xiaoming Lu, Jishuai Su, Yongfei Bai

**Affiliations:** State Key Laboratory of Vegetation and Environmental Change, Institute of Botany, Chinese Academy of Sciences, Beijing 100093, China; College of Life Sciences, University of Chinese Academy of Sciences, No. 19A Yuquan Road, Beijing 100049, China; State Key Laboratory of Vegetation and Environmental Change, Institute of Botany, Chinese Academy of Sciences, Beijing 100093, China; College of Life Sciences, University of Chinese Academy of Sciences, No. 19A Yuquan Road, Beijing 100049, China; State Key Laboratory of Vegetation and Environmental Change, Institute of Botany, Chinese Academy of Sciences, Beijing 100093, China; College of Life Sciences, University of Chinese Academy of Sciences, No. 19A Yuquan Road, Beijing 100049, China; State Key Laboratory of Vegetation and Environmental Change, Institute of Botany, Chinese Academy of Sciences, Beijing 100093, China; College of Resources and Environment, University of Chinese Academy of Sciences, No. 19A Yuquan Road, Beijing 100049, China

**Keywords:** indigenous soil microbiota, phosphorus addition, planting pattern, defence genes, transcriptome sequencing, monoculture, mixture, semiarid steppe, *Cleistogenes squarrosa*, *Leymus chinensis*

## Abstract

**Background and Aims:**

Growing evidence has suggested that plant responses to model soil microorganisms are context dependent; however, few studies have investigated the effects of whole soil microbial communities on plant performance in different abiotic and biotic conditions. To address this, we examined how soil phosphorus (P) availability and different planting patterns regulate soil microbial effects on the growth of two native plant species in a semiarid steppe.

**Methods:**

We carried out a glasshouse experiment to explore the effects of the whole indigenous soil microbiota on the growth and performance of *Leymus chinensis* and *Cleistogenes squarrosa* using soil sterilization with different soil P availabilities and planting patterns (monoculture and mixture). Transcriptome sequencing (RNA-seq) was used to explain the potential molecular mechanisms of the soil microbial effects on *C. squarrosa*.

**Key Results:**

The soil sterilization treatment significantly increased the biomass of *L. chinensis* and *C. squarrosa* in both monoculture and mixture conditions, which indicated that the soil microbiota had negative growth effects on both plants. The addition of P neutralized the negative microbial effects for both *L. chinensis* and *C. squarrosa*, whereas the mixture treatment amplified the negative microbial effects on *L. chinensis* but alleviated them on *C. squarrosa*. Transcriptomic analysis from *C. squarrosa* roots underscored that the negative soil microbial effects were induced by the upregulation of defence genes. The P addition treatment resulted in significant decreases in the number of differentially expressed genes attributable to the soil microbiota, and some defence genes were downregulated.

**Conclusions:**

Our results underline that indigenous soil microbiota have negative effects on the growth of two dominant plant species from a semiarid steppe, but their effects are highly dependent on the soil P availability and planting patterns. They also indicate that defence genes might play a key role in controlling plant growth responses to the soil microbiota.

## INTRODUCTION

The soil microbiota is a key driver of plant growth, community structures and ecosystem functions (Wardle *et al.*, 2004; Schnitzer *et al.*, 2011; Wagg *et al.*, 2014). Many previous studies have focused on the effects of a single model microorganism on plants, which is a rare situation in nature, whereas increasing evidence has demonstrated that it is the whole indigenous soil microbiota that is more important for plant growth and performance (Li *et al.*, 2019; Wei *et al.*, 2019; Trivedi *et al.*, 2020). For example, the functions of indigenous soil microbiota related to nutrient cycle and plant defence strategies are decisive for the successful ecological restoration of degraded ecosystems (Bargali *et al.*, 2019; Awasthi *et al.*, 2022; Manral *et al.*, 2022), because they directly affect the plant community composition and soil health (Pant *et al.*, 2017; Manral *et al.*, 2020). Soil sterilization is a common approach to estimating the net effects of the whole soil microbiota on plant performance, especially for studies on crop replanting diseases in agroecosystems (Qiao *et al.*, 2022). However, the responses of plant species in a natural community to the indigenous soil microbiota are constantly changing, which is attributable, in part, to high levels of resource competition (Yang *et al.*, 2015). Therefore, a better understanding of the soil microbial effects on dominant plant species in a natural system and how they are mediated by abiotic (e.g. soil nutrient availability) and biotic factors (e.g. neighbouring plants) is essential to development, restoration and management.

The indigenous soil microbiota affects plant growth both positively and negatively (van der Heijden *et al.*, 2008). If the entire microbiota has a positive growth effect on plants, this indicates that beneficial microbiota plays a governing role over pathogenic microbiota, whereas a negative growth effect shows that pathogens are more powerful (van der Putten, 2017). The activation of plant defence systems might unravel the biological mechanisms underlying the effect of soil microbiota (Mathesius, 2009). For example, accumulation of soil pathogens can reduce a surge of reactive oxygen species, release a quorum sensing signal and increase metabolic enzyme activities, and this will simultaneously stimulate defence gene expression in plants (Rey *et al.*, 2019; Singh *et al.*, 2022). However, the trade-off between the beneficial microbiota and pathogens is altered by environmental variables, and the balance can be disturbed by changing conditions (i.e. soil nutrient levels and plant diversity). For example, neutral to positive soil microbial effects are found to be dominant when soil fertility is low, whereas negative soil microbial effects occur as soil fertility increases (Luo *et al.*, 2017). Recent studies have also demonstrated that plants suffer more from specific pathogens in a community with low plant diversity and that the impacts of pathogens reduce as plant richness increases (Kulmatiski *et al.*, 2012; Mommer *et al.*, 2018; Thakur *et al.*, 2021). It is therefore imperative that we understand the key abiotic and biotic factors regulating the effects of the microbiota on plants.

Among the abiotic factors, phosphorus (P) is not only one of the most limited nutrient elements controlling plant growth in natural ecosystems but is also a vital regulator between plants and soil microbiota (Bever *et al.*, 2010; Cordell and White, 2014; Padalia *et al.*, 2022). On the one hand, variation in soil P availability can change the soil biota biomass and community composition (Huang *et al.*, 2016; Ikoyi *et al.*, 2020) and, thereby, directly determine plant–microbiota interactions. On the other hand, soil P availability can affect plant growth and carbon (C) allocation, which indirectly impacts the plant–microbiota interactions. For example, with increasing soil P availability, plants allocate less C to the mycorrhizal fungi (Ji and Bever, 2016), the symbiosis of plants with arbuscular mycorrhizal fungi (AMF) changes from mutualism to parasitism (Johnson, 2010), and the expression of genes encoding PHO84 (the phosphate transporter protein) decreases (Ren *et al.*, 2022). Moreover, high P availability might enhance plant defence capabilities against certain soil pathogens (Malhotra *et al.*, 2018) and influence defence gene expression (Lin *et al.*, 2013). Hence, plants can alter their interactions with the soil microbiota to maintain their own P homeostasis (Li *et al.*, 2016), and soil P availability determines the subsequent soil microbial effects (Mezeli *et al.*, 2020).

Among the biotic factors, the planting pattern is a crucial factor to control soil microbial effects. Plants and soil microbiota are influenced and selected by each other (Berendsen *et al.*, 2012), resulting in a variable net microbial effect. Considerable evidence has suggested that the soil microbiota can induce species-specific effects on plant growth (Bennett *et al.*, 2017; Teste *et al.*, 2017), especially for pathogens (Bever *et al.*, 2015). However, the responses of different plant species to the same microbial inoculation can vary considerably (Zhou *et al.*, 2018). Klironomos (2002) found that invasive plant species exhibit positive feedback with their ‘home soil’, whereas rare plant species exhibit negative feedback. Plant mixtures could also adjust the relationships between plant species and microorganisms and could even help to decrease the abundance of certain pathogens for their host plants (Mommer *et al.*, 2018). For example, with increasing plant diversity, the negative effects of pathogens on host plant species can be diminished and the positive growth effects caused by the AMF can be strengthened (Maron *et al.*, 2011; Zhang and Feng, 2021). Taking account of the soil P availability, researchers have found that P limitation can benefit the coexistence of species more than P sufficiency with the presence of soil microbiota (Olde Venterink, 2011; Ceulemans *et al.*, 2014, 2017). This leads us to question how soil P availability and neighbouring plants regulate soil microbial effects at the same time.

Soil sterilization is widely regarded as the ultimate method to examine the functions of the whole soil microbiota, because it kills all creatures without discrimination. Common soil sterilization methods include autoclaving, chemical fumigation and γ-irradiation for laboratory experiments (Troelstra *et al.*, 2001; Cha *et al.*, 2019; Wang *et al.*, 2021), all of which will, inevitably, have an adverse effect on soil properties (Razavi and Lakzian, 2007). Owing to its pollution-free and high-efficiency characteristics, γ-irradiation is considered to be a comparatively more effective and superior approach to sterilization, with a lower impact on the soil physical structure and nutrient levels than other common soil sterilization methods (McNamara *et al.*, 2003; Berns *et al.*, 2008; Wehr and Kirchhof, 2021). Gama-irradiation results in soil microbial cell lysis and nutrient release, which ineluctably affects soil nitrate and ammonium concentrations (Buchan *et al.*, 2012). Hendriks *et al.* (2013) demonstrated that mineral nitrogen (N) released by soil sterilization did not influence the effects of soil biota on plant growth. Yu *et al.* (2019) observed a marked reduction in growth and nutrients in plants cultivated in sterilized soil and inferred that nutrients released by sterilization might be too low to improve plant growth. Therefore, soil sterilization by γ-irradiation is accepted as an ideal method to study the soil microbiota.

As noted above, it is still poorly understood how indigenous soil microbiota affect plant performance in different biotic and abiotic conditions. In this study, we conducted a glasshouse experiment to explore the effects of indigenous soil microbes on the growth of *Leymus chinensis* and *Cleistogenes squarrosa* from a semiarid steppe in Inner Mongolia, China, and examined how soil microbial effects are regulated by soil P availability and different planting patterns. The semiarid steppe in Inner Mongolia is an essential part of the Eurasian steppe that has been widely disturbed by global climate change and anthropogenic activities. Exploring indigenous soil microbial functions and how they are affected by environmental changes will provide valuable information to aid in grassland conservation and restoration (Zhang *et al.*, 2021). *L. chinensis* is a dominant perennial C_3_ rhizome grass of the Eurasian steppe, whereas *C. squarrosa* is not only the dominant perennial C_4_ bunchgrass, but also an indicator species of grassland degradation (Bai *et al.*, 2012). In addition, *L. chinensis* and *C. squarrosa* adopt different ecological strategies in terms of resource acquisition and growth rate. Specifically, *L. chinensis* exhibits an acquisitive strategy, with high foliar nutrient content, large leaf area and high specific root length related to a high ability to acquire resources, fast tissue turnover and fast growth rate (Zheng *et al.*, 2010). In contrast, *C. squarrosa* has a conservative strategy, with functional traits related to low leaf nutrient content, small leaf area and low ability to capture resources (Tecco *et al.*, 2010; Zheng *et al.*, 2010). Our experiment included soil sterilization, P addition and different planting patterns (monoculture and mixture) for *L. chinensis* and *C. squarrosa*. Specifically, we addressed three research questions. First, how do *L. chinensis* and *C. squarrosa* respond to the presence and absence of indigenous soil microbiota? Second, how do P additions and different planting patterns (monoculture and mixture) regulate soil microbial effects on *L. chinensis* and *C. squarrosa*? Third, what is the molecular mechanism of the soil microbial effects on *C. squarrosa*, and how do P additions affect defence gene expression? We hypothesized that: (1) indigenous soil inoculation will suppress plant growth, because negative plant–soil feedbacks are widespread in grassland ecosystems (Petermann *et al.*, 2008) and graminoid–microbial interactions are particularly negative (Li *et al.*, 2020); (2) P additions and plant mixtures will enhance plant growth, which can reduce the growth inhibition of indigenous microbiota to some extent; and (3) soil microbiota can induce the plant immunity system and upregulate defence genes.

## MATERIALS AND METHODS

### Study site and field sampling

The study was based at the Inner Mongolia Grassland Ecosystem Research Station (IMGERS; 116°42ʹE, 43°38ʹN) of the Chinese Academy of Sciences, located in the Inner Mongolia Autonomous Region of China (Bai *et al.*, 2004). The vegetation type is that of a typical steppe, and the soil is classified as dark chestnut. The area has a temperate semiarid continental climate, with a mean annual precipitation of 346.1 mm. The mean annual temperature is 0.3 °C, with the coldest (January) mean temperature being −21.6 °C and the hottest (July) being 19.0 °C. The dominant plant species at the study site are *L. chinensis*, *C. squarrosa*, *Stipa grandis*, *Agropyron cristatum*, *Carex korshinskii* and *Achnatherum sibiricum* (Bai *et al.*, 2004).

Soil samples for inoculation were collected from a natural steppe community in August 2017, which had been fenced from grazing since 1979. Specifically, soil samples were collected from five 1 m × 1 m plots by using the five-point sampling method (Jin *et al.*, 2015). In each plot, five soil columns were excavated from the topsoil layer (0–20 cm), where the plant community was dominated by *L. chinensis* or *C. squarrosa*. After removal of the plant coarse roots and litter by using a 2 mm sieve, the soil was mixed with fine root segments retained, and stored in a refrigerator at 4 °C until further inoculation.

Seeds of *L. chinensis* and *C. squarrosa* were also collected in August 2017 from the IMGERS. When seeds matured, which started in mid-August, the seed heads were immediately clipped from the plants and allowed to dry for 2 weeks. Seeds were then stripped off the spikelet and left to dry before being stored in a cool and shady place for 1 month. Seeds of *L. chinensis* were soaked in distilled water for 48 h to break their dormancy before germination.

### Experimental design

The experiment used a completely randomized design with three factors: two soil treatments (sterilized inoculation soil and non-sterilized inoculation soil; sterilization was via 25 kGy γ-irradiation), two levels of P addition (5 and 25 mg kg^−1^ addition in vermiculite, equivalent to 12 and 60 kg ha^−1^ P additions at the field site, respectively, and designated as the low and high P treatments, respectively) and three planting patterns (*C. squarrosa* monoculture, *L. chinensis* monoculture, and a mixture of *C. squarrosa* and *L. chinensis*). Each treatment combination was replicated seven times, resulting in a total of 84 pots.

To explore, from a molecular aspect, the mechanisms controlling regulation of the plant microbial effects in response to the P addition, we also conducted a transcriptome-sequencing (RNA-seq) experiment at the same time. We chose to use *C. squarrosa* to explore the molecular mechanisms of the soil microbial effect. This is because gene expressions within *L. chinensis* rhizomes are inherently different from those of roots (Chen *et al.*, 2013), which would be more obscure if we were to compare gene expressions of rhizomes and roots of *L. chinensis* with roots of *C. squarrosa*. In addition, it is inappropriate to compare the gene annotation expression levels within *L. chinensis* and *C. squarrosa*, both of which are non-model plants lacking reference genomes (Ockendon *et al.*, 2016). For this experiment, we planted *C. squarrosa* in monoculture, using the two P levels and two soil sterilization treatments previously described. Each treatment combination was replicated three times, resulting in a total of 12 pots for RNA-seq sample collection.

### Growth conditions

Seeds were surface disinfected using 70 % alcohol before germination. Seeds were sown in Petri dishes 2–3 weeks before transplantation. Individuals that showed consistent growth were selected for transplantation. We used 25 kGy γ-irradiation to sterilize the plant growth substrates and inoculated soil samples. The pot had a capacity of ~4.5 L, and we used vermiculite, sand and indigenous soil as the plant substrate. At the bottom of each pot, 300 g of sand was placed for drainage, followed by 950 g of vermiculite and 300 g of sterilized or non-sterilized inoculation soil. Finally, 150 g of sterilized vermiculite was placed on the surface to hinder the evaporation of the soil water and airborne contamination. The growth medium was a 1:2:14 (v:v:v) mixture of sand, indigenous soil inoculation and vermiculite. The mixture was stratified as already described during the growth period and mixed well during harvest for measurement. For the monoculture treatment, two individuals of the same species were planted in each pot, and for the mixture treatment, one individual of each species was planted in each pot. Phosphorus was added in the Na_2_HPO_4_ solution. To ensure the supply of other nutrients, 100 mg kg^−1^ N (NH_4_NO_3_) and 200 mL refined Hoagland solution, which contained 607 mg L^−1^ K_2_SO_4_, 493 mg L^−1^ MgSO_4_, 66 mg L^−1^ NH_4_H_2_SO_4_, 20 mg L^−1^ Fe(Na)EDTA,15 mg L^−1^ FeSO_4_, 2.86 mg L^−1^ H_3_BO_3_, 2.13 mg L^−1^ MnSO_4_, 0.05 mg L^−1^ CuSO_4_ and 0.22 mg L^−1^ ZnSO_4_, were added to each pot.

All pots were placed in the glasshouse at the Institute of Botany, the Chinese Academy of Sciences, from November 2017 to March 2018, with day/night temperatures of 25/18 °C, with a 16 h–8 h light–dark cycle and 60 % relative humidity. Supplemental lighting was used from 06:00 to 20:00 at 400–600 μmol m^−2^ s^−1^ light intensity to increase illuminance. No pesticides were used during the experiment. The pots were watered such as to maintain 65 % of the field holding water content every 4 days. The positioning of the pots was changed every week to eliminate differences in growth conditions.

### Plant and soil harvest

Plants were grown for 16 weeks in the glasshouse, after which the roots, stems, leaves and soil were harvested. The plant samples were oven-dried at 75 °C for 72 h to measure the dry weight. We randomly chose three replicates for each of the plant and soil samples to measure the C, N and P contents.

The soil and plant tissue P concentrations were determined using inductively coupled plasma-optical emission spectrometry (ICAP 6300, Thermo Scientific, USA), and soil C and N concentrations were measured using an elemental analyser (Vario EL III; Elementar, Germany). We used the colorimetric method to assess the soil available P content via NaHCO_3_ digestion with an ultraviolet–visible spectrophotometer (UV-2550; Shimadzu, Japan). Soil NH_4_^+^ and NO_3_^−^ concentrations were extracted using 2 m KCl and determined using a flow injection autoanalyser (SEAL Analytical, Germany). Soil pH was measured with a pH meter (FE20-FiveEasy; Mettler Toledo, Switzerland). S-ACP and S-AKP protocol kits were used to measure soil acid phosphatase (ACP) and soil alkaline phosphatase (AKP) activity, respectively, by following the manufacturer’s instructions (Solarbio, Beijing, China) (Shao *et al.*, 2020; Li *et al.*, 2021). The AMF colonization rate was determined using the procedure adopted by Phillips and Hayman (1970). Briefly, 40 cleaned root fragments ~1 cm in size were cleared with 10 % KOH in a 90 °C water bath for 50 min, kept in 2 % HCl solution for 10 min, and stained with 0.05 % Trypan Blue for 30 min. Lactic acid–glycerol solution was used for destaining and preservation.

### Plate tests

A serial dilution plate test was used to assess the effect of sterilization with seven dilutions ranging from 10^−1^ to 10^−7^ times. Indigenous soil, sand and vermiculite were tested alone. Five grams of soil substrate was mixed with 45 mL of sterilized water and shaken for 20 min. The solution was diluted 10-fold. Three types of culture media were used to ensure the comprehensive primary recovery of the soil microbiota: Luria broth medium for growing bacteria; potato dextrose agar medium for growing fungi; and Gauze’s medium No. 1 for growing actinomycetes. Plates were inverted and incubated at 37 °C for 7 days to observe the microbial colonies.

### Transcriptome-sequencing analysis

Intact root systems were removed from the plants, shaken gently to remove their soil, and washed quickly. The root elongation areas were selected as samples, covered with tinfoil, then immediately frozen in liquid N and stored at −80 °C. Total RNA was extracted from the roots using the Trizol reagent (Invitrogen, CA, USA) following the manufacturer’s recommendations, and the RNA purity was checked using a NanoPhotometer spectrophotometer (IMPLEN, CA, USA). Briefly, for each sample, a total of 1.5 μg RNA was used as the input material for RNA sample preparations. The mRNA was fragmented into short fragments using a fragmentation buffer. We used mRNA as the template to synthesize repaired cDNA. To select cDNA fragments that were 250–300 bp in length, the library fragments were purified with the AMPure XP system (Beckman Coulter, Beverly, MA, USA). PCR was performed using Phusion High-Fidelity DNA polymerase, random hexamer primers, and Index (X) Primer. The PCR products were then purified (AMPure XP system), and the library quality was assessed using the 2100 Bioanalyzer system (Agilent Technologies, CA, USA). The cDNA libraries were sequenced on an Illumina Hiseq platform, and paired-end reads were generated.

Raw data (raw reads) in the fastq format were filtered by removing reads containing adapter, reads with Ploy-N and low-quality reads. At the same time, the Q20, Q30, guanine–cytosine (GC) content and sequence duplication levels of the clean data were calculated.

Transcriptome assembly was accomplished using Trinity (Grabherr *et al.*, 2011), after which the unigenes were obtained. Unigene functions were annotated based on the following databases: KOG (Clusters of eukaryotic Orthologous Groups of proteins; https://www.ncbi.nlm.nih.gov/research/cog-project/) and KO (KEGG Ortholog database; http://www.genome.jp/kegg/).

The expected number of fragments per kilobase of transcript sequence per millions of base pairs sequenced (FPKM) method was used to calculate the unigene expression levels. Differential expression analysis was performed using the DESeq2 R package (Love *et al.*, 2014). The resulting *P*-values were adjusted using Benjamini and Hochberg’s approach to control the false discovery rate. An adjusted *P*-value < 0.05 and |log_2_FoldChange| ≥ 2 were assigned as thresholds to adjudicate the significance of differential gene expression. KOBAS software was used to test the statistical enrichment of differential gene expression in the KEGG pathways (Xie *et al.*, 2011). All raw RNA-seq data have been uploaded to the NCBI database (https://www.ncbi.nlm.nih.gov/sra) under sequence read archive SRP302089 (Bioproject ID: PRJNA692693).

### Statistical analysis

Microbial effects were calculated according to eqn (1), which was modified from Brinkman *et al.* (2010):


$$\begin{array}{l} Microbial\;\;\;effects\;=\;ln\left(\frac{NS}{S_{mean}}\right) \end{array}$$ (1)

where NS represents the total dry weight of plants in the soil non-sterilization treatment, and *S*_mean_ is the averaged total dry weight of the plants in the soil sterilization treatment. Positive values of the microbial effects showed that plant growth was enhanced by soil microbiota, and negative values showed that plant growth was inhibited by soil microbiota.

All statistical analyses were conducted using R v.4.0.4 (R Core Development Team, 2021). Three-way ANOVA was performed to analyse the effects of three factors (soil sterilization, P addition and planting pattern) on plant biomass, plant P concentration and soil properties. The Shapiro–Wilk test was used to assess the normality of the data and Levene’s test to assess the homogeneity of variance. Given that the total sample sizes for plant biomass and soil properties were both >30, with an equal subsample size, there were no alternative non-parametric tests, and three-way ANOVA was still a robust statistical procedure, although minor assumptions were violated (Ghasemi and Zahediasl, 2012; Howell, 2013). For plant P concentrations, normality and homogeneity of variance were checked before three-way ANOVA. The total P concentration of *L. chinensis* was ln-transformed to achieve normality and homogeneity of variance. Two-way ANOVA was used to assess the microbial effects, and data of the *L. chinensis* microbial effects were sin-transformed to achieve normality and homogeneity of variance (Supplementary data Table S1). A *t*-test was conducted to evaluate changes in soil substrate nutrients before and after γ-irradiation. Cohen’s *d* was used to estimate the effect size for two-sample *t*-tests in multiple comparisons, and partial η^2^ was used to estimate the effect size for ANOVA (Fritz *et al.*, 2012; Lakens, 2013).

## RESULTS

### Assessment of the effect of soil sterilization

No microbial colonies were observed in plate tests with any of the culture media used with the sterilization treatment, whereas microbial colonies were detected to varying degrees with the non-sterilized treatment (Supplementary data Fig. S1).

The soil sterilization treatment significantly increased the concentrations of total C, N, P, available P and NH_4_-N in the indigenous soil. The C (%), N (%) and the mineral N in the sand and vermiculite were too low to detect using the elemental analyser. The sterilization treatment significantly reduced the available P concentration for the vermiculite and NO_3_-N of the indigenous soil (Supplementary data Table S2).

### Plant biomass

**Fig. 1.**
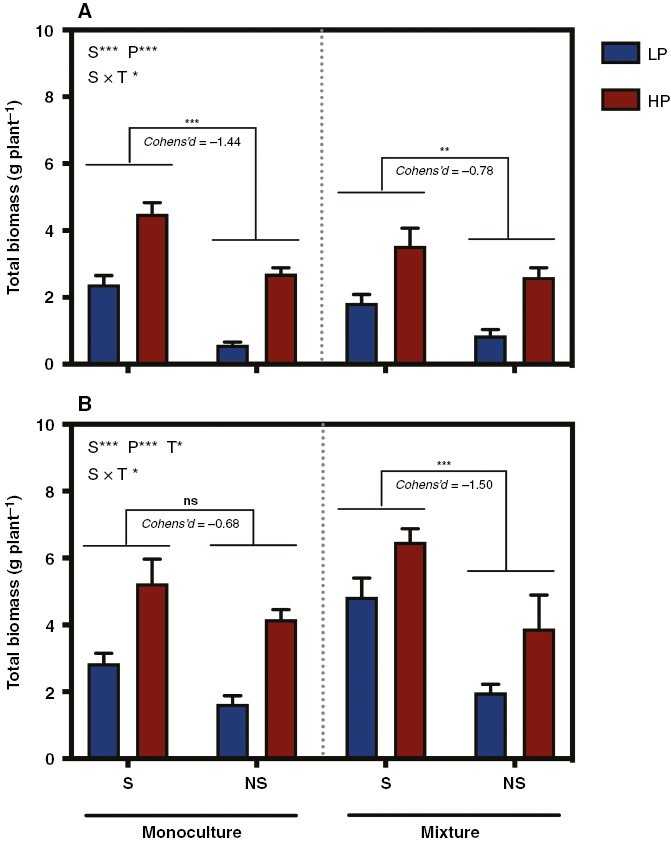
Effects of P additions and soil sterilization on the total biomass of monocultures and mixtures of *Cleistogenes squarrosa* (A) and *Leymus chinensis* (B). Bars show the means + s.e.m. (*n* = 7). *P < 0.05, **P < 0.01 and ***P < 0.001; ns represents no significant effects at *P* < 0.05. Abbreviations: HP, high P; LP, low P; NS, soil non-sterilization; P, phosphorus level; S, soil sterilization; T, planting pattern.

For *C. squarrosa*, the soil sterilization and P addition treatments both significantly increased the shoot and root biomass (Table 1; Supplementary data Fig. S2). Planting pattern and soil sterilization had a significant interactive effect on total biomass. Specifically, soil sterilization increased the total biomass more in the monoculture than in the mixture (Table 1; Fig. 1A; |Cohen’s *d*| = 1.44 in the monoculture; |Cohen’s *d*| = 0.78 in the mixture).

**Table 1.** *Results (*F*-values) of the three-way ANOVA for effects of soil sterilization (S), P level (P) and planting pattern (T) and their interactions with plant shoot, root and total biomass*

	Cleistogenes squarrosa			Leymus chinensis		
Source	Shoot biomass	Root biomass	Total biomass	Shoot biomass	Root biomass	Total biomass
S	35.30***	41.22***	46.25***	29.29***	12.60***	26.19***
P	92.01***	19.45***	90.41***	26.45***	23.20***	31.43***
T	1.85	3.46	2.73	7.56*	0.99	4.76*
S × P	0.06	0.42	0.00	1.51	0.87	0.07
S × T	3.19	4.30	4.32*	8.03*	0.51	4.36*
P × T	0.88	0.42	0.96	1.09	0.27	0.81
S × P × T	0.02	0.50	0.00	0.03	0.14	0.01

*P < 0.05 and ***P < 0.001.

For *L. chinensis*, as with *C. squarrosa*, the soil sterilization and P addition treatments both significantly increased the shoot and root biomass (Table 1; Supplementary data Fig. S2). Moreover, the planting pattern had a significant effect on the total biomass and shoot biomass: biomass was lower in the monoculture than in the mixture. The planting pattern and soil sterilization also had a significant interactive effect on the total biomass and shoot biomass. Specifically, soil sterilization significantly increased these variables in the mixture but had no significant effects on the monoculture, suggesting that the mixture treatment magnified the negative effects of the soil microbiota on the total biomass (Table 1; Fig. 1B).

### Phosphorus concentration

**Fig. 2.**
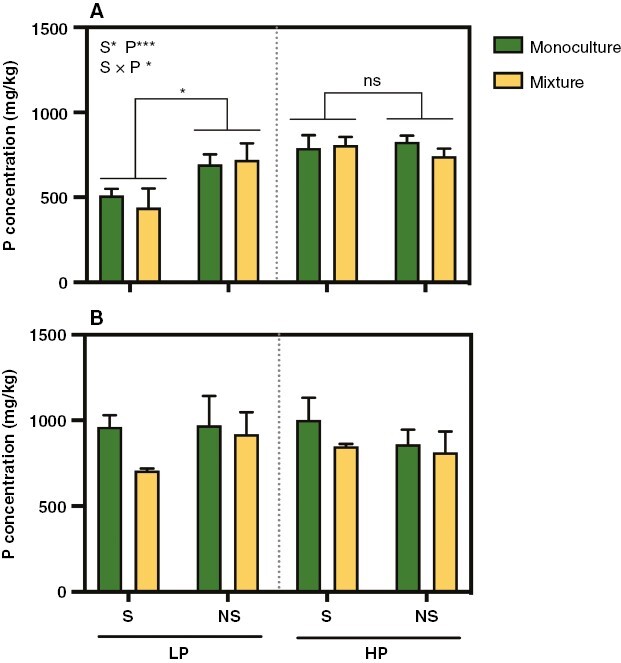
Effects of P addition and soil sterilization on the whole plant P concentration (in milligrams per kilogram) in monocultures and mixtures of *Cleistogenes squarrosa* (A) and *Leymus chinensis* (B). Bars show the means + s.e.m. (*n* = 3). Abbreviations: HP, high P; LP, low P; NS, soil non-sterilization; P, phosphorus level; S, soil sterilization. *P < 0.05 and ***P < 0.001; ns represents no significant effects at *P* < 0.05. Insignificant groups are not identified.

For *C. squarrosa*, the soil sterilization treatment significantly decreased the total P and shoot P concentrations, with these variables being higher in the non-sterilized treatment than in the sterilized treatment. Addition of P significantly increased the total, shoot and root P concentrations (Fig. 2A; Supplementary data Table S3; Fig. S3). In addition, the soil sterilization and P addition treatments had significant interactive effects on the total P and shoot P concentrations. The soil sterilization significantly decreased the P concentration under low P, whereas it had no significant effect on the P concentration with the addition of P (Fig. 2A; Supplementary data Table S3; Fig. S3). Planting pattern had no primary or interactive effect on the P concentration (Fig. 2A).

For *L. chinensis*, the soil sterilization, P addition and planting pattern all had no significant or interactive effects on the shoot, root and total P concentrations (Fig. 2B; Supplementary data Table S3; Fig. S3).

### Microbial effects

**Fig. 3.**
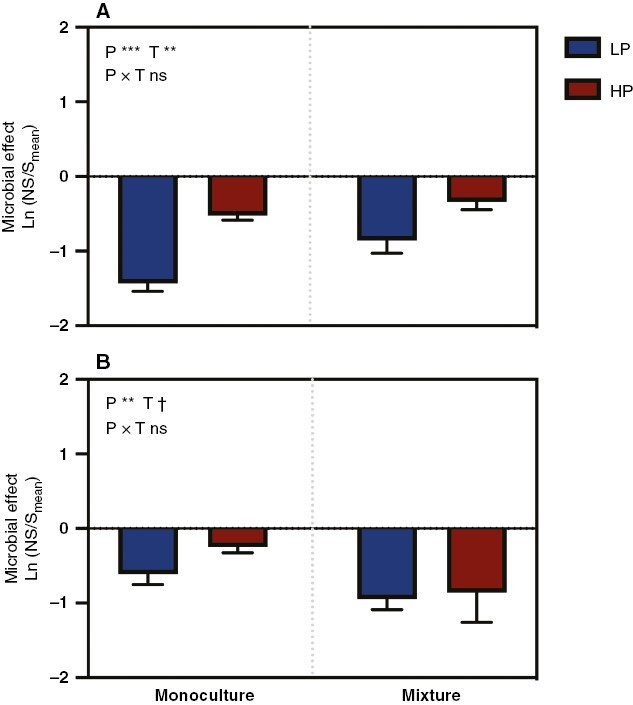
Effects of P addition and planting pattern on the microbial effects of *Cleistogenes squarrosa* (A) and *Leymus chinensis* (B). Microbial effects are calculated using the total biomass. Bars show the means ± s.e.m. (*n* = 7). **†***P* < 0.1, **P < 0.01 and ***P < 0.001; ns represents no significant effects at *P* < 0.1. Abbreviations: HP, high P; LP, low P; NS, soil non-sterilization; P, phosphorus level; S, soil sterilization; T, planting pattern.

The soil microbial effects were all observed to be negative for the growth of the two plant species in all treatments (Fig. 3). For *C. squarrosa*, the P addition and planting pattern treatments reduced the negative effects caused by the soil microbiota on biomass. The P addition had a stronger effect when compared with the mixture treatment, because the partial η^2^ for the P addition was larger than that for the planting pattern (Fig. 3A; Supplementary data Table S4; η^2^ = 0.59 for P addition and η^2^ = 0.29 for planting pattern). For *L. chinensis*, the addition of P also significantly mitigated the negative microbial effect, but the mixture treatment aggravated the negative microbial effect (*P* < 0.1; Fig. 3B; Supplementary data Table S4).

### Soil phosphatase activities, pH and AMF colonization rate

**Fig. 4.**
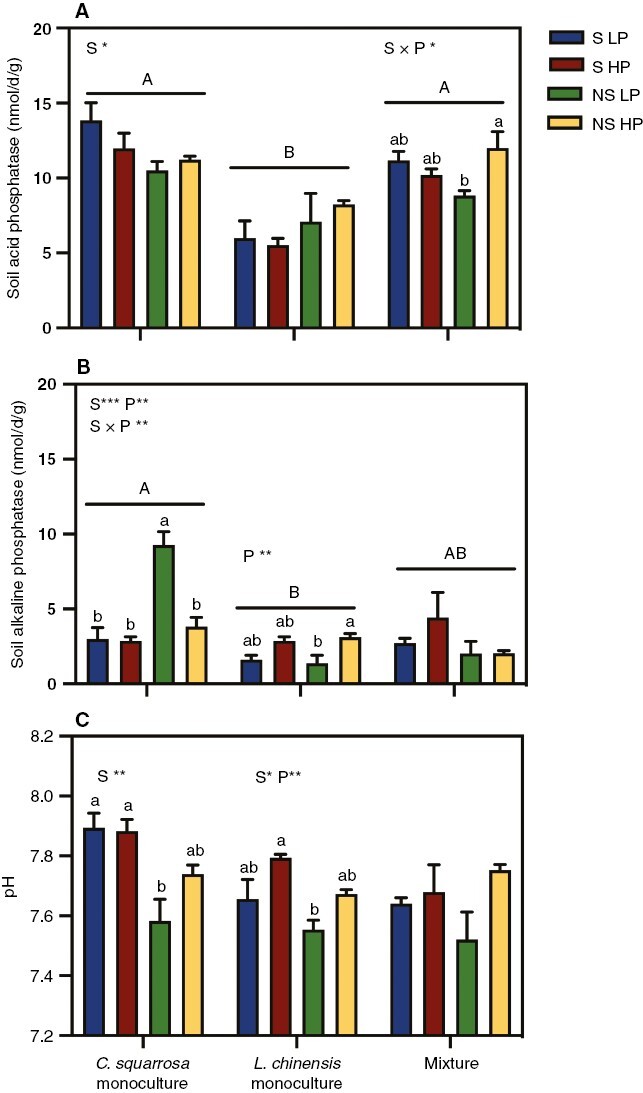
Responses of soil acid phosphatase activity (A), soil alkaline phosphatase activity (B) and soil pH (C) to P addition and soil sterilization in monocultures and mixtures of *Cleistogenes squarrosa* and *Leymus chinensis*. Bars show the means ± s.e.m. (*n* = 3). Different uppercase letters indicate significant differences among planting patterns (one-way ANOVA, *P* < 0.05). For each planting pattern, the effects of the P addition and soil sterilization are shown (two-way ANOVA, *P < 0.05, **P < 0.01 and ***P < 0.001). Insignificant groups are not identified. Within each planting pattern, means with different lowercase letters are significantly different (Tukey’s HSD, *P* < 0.05). Abbreviations: HP, high P; LP, low P; NS, soil non-sterilization; S, soil sterilization.

The planting pattern had a significant effect on the ACP activity, AKP activity and soil pH. Specifically, the ACP and AKP activities were higher in the *C. squarrosa* monoculture than in the *L. chinensis* monoculture (Fig. 4A, B; Supplementary data Table S5). For the *C. squarrosa* monoculture, the soil sterilization treatment had significant effects on the ACP activity, AKP activity and soil pH. Specifically, soil sterilization significantly lowered the AKP activity and increased the ACP activity and pH (Fig. 4). In addition, the soil sterilization treatment and P addition treatments had significant interactive effects on the AKP activity, with AKP activity being highest in the non-sterilized soil combined with low P treatment (Fig. 4B). For the *L. chinensis* monoculture, the P addition treatment significantly affected AKP activity and pH. Specifically, the P addition significantly increased AKP activity and pH but had no significant effect on ACP activity (Fig. 4). The soil sterilization treatment also significantly affected pH, because the pH was higher in the sterilized treatment than in the non-sterilized treatment (Fig. 4C). For the mixture, soil sterilization and P addition had a significant interactive effect on the ACP activity: P addition significantly increased ACP activity in the non-sterilized treatment but had no significant effects on the sterilized treatment (Fig. 4A).

The sterilized treatment was free of AMF colonization, and the mean AMF colonization rates were all <15% in the non-sterilized treatment and had no significant effects between the treatments (Fig. S4; Supplementary data Table S6).

### Transcriptome sequencing

**Fig. 5.**
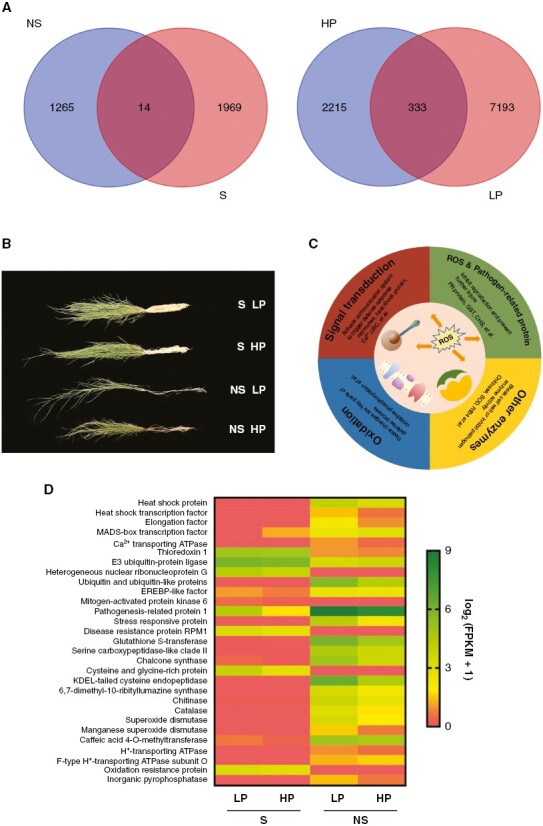
Transcriptome sequencing (RNA-seq) analysis of *Cleistogenes squarrosa* roots from the soil sterilization and P addition treatments. (A) Venn diagram showing the number of overlapping and non-overlapping differentially expressed genes for LP vs. HP and for NS vs. S. (B) Morphological differences of *C. squarrosa* under various treatments. (C) Classification of the defence genes detected in our study. Defence genes were divided into four catgories: signal transduction; reactive oxygen species (ROS) and pathogen-related proteins; enzymes; and oxidation. (D) Expression pattern of the defence genes in *C. squarrosa* roots. The expression values were normalized by log_2_(FPKM + 1) to eliminate the effect of the zero value. Abbreviations: CHS, chalcone synthase; GST, glutathione *S*-transferase; HP, high P level; LP, low P level; NS, soil non-sterilization; PR protein, pathogenesis-related protein; RIB4, 6,7-dimethyl-8-ribityllumazine synthase; SOD, superoxide dismutase; UBC, ubiquitin C; S, soil sterilization.

As already mentioned, the P addition and mixture treatments could both alleviate the negative soil microbial effect on the growth of *C. squarrosa*, and the effect was stronger with the P addition (Figs 3A and 5B; Supplementary data Table S4). Here, RNA-seq was used to test the molecular mechanisms of the soil microbial effect on the *C. squarrosa* with different levels of P addition. A total of 422 327 unigenes were obtained, which were annotated using the KOG and GO databases. We found that 1983 differentially expressed genes (DEGs) responded to the P addition treatment combined with the soil sterilization treatment and 1279 to the P addition treatment with the non-sterilized treatment. For the P addition treatment, 7526 DEGs responded to soil sterilization with the low P addition and 2548 to soil sterilization with the high P addition (Fig. 5A).

The DEGs that responded to the soil sterilization treatment overlapped between the low and high P addition treatments, and these overlapping DEGs were classified as soil microbiota-specific genes. Three hundred and thirty-three soil microbiota-specific genes (Fig. 5A) were obtained, of which 164 genes were annotated in the KEGG or KOG database. Excluding genes enriched in the ribosome, we found 44 unigenes that were attributed to defence (Supplementary data Table S7). We divided these genes into four catgories: signal transduction, reactive oxygen species and pathogen-related proteins, other enzymes, and oxidation (Fig. 5C; Supplementary data Table S7).

Interestingly, genes encoding the heat shock proteins, Ca^2+^-transporting ATPase, ubiquitin and ubiquitin-like proteins, pathogen-related proteins, stress-responsive protein, cysteine endopeptidase, 6,7-dimethyl-8-ribityllumazine synthase, chitinase, catalase, glutathione *S*-transferase, chalcone synthase and genes involved in oxidative phosphorylation were upregulated in the soil non-sterilization treatment. A few genes, encoding thioredoxin 1, E3 ubiquitin-protein ligase, mitogen-activated protein kinases and disease-resistance protein RPM1, were downregulated (Fig. 5D; Supplementary data Table S7).

Notably, expressions of defence genes, which were induced by soil microbiota, were decreased with the P addition treatment, such as those of ubiquitin and ubiquitin-like proteins, pathogen-related proteins, stress-responsive protein, chalcone synthase, cysteine endopeptidase, 6,7-dimethyl-8-ribityllumazine synthase, chitinase, catalase and glutathione *S*-transferase (Fig. 5D; Supplementary data Table S6). Moreover, except for genes involved in defence and ribosome pathways, the expression of all upregulated soil microbiota-specific genes decreased after P addition (Supplementary data Table S8). For example, genes encoding serine carboxypeptidase, protein disulphide-isomerase A1, inorganic phosphate, histone H2B, actin, 6-phosphogluconate dehydrogenase, alcohol dehydrogenase and sorbitol dehydrogenase were upregulated by the soil microbiota but had lower levels of expression when P was added (Supplementary data Table S8).

## DISCUSSION

### Effects of soil sterilization

Our results demonstrated that soil sterilization could significantly promote the plant biomass of both *L. chinensis* and *C. squarrosa*, regardless of the planting pattern. There are several reasons that could explain these results. First, these findings could be related to the fact that soil pathogens are important agents that lead to biomass reduction (Bever, 1994; Bever *et al.*, 1997). For example, more pathogenic fungal operational taxonomic units can be found in monocultures, and these pathogens can have species-specific detrimental impacts on plant growth (Kulmatiski *et al.*, 2012; Mommer *et al.*, 2018). The restrictions on plant growth caused by pathogens could thus be relieved after soil sterilization, and the plant biomass could be accumulated. Most pathogens in the inoculated indigenous soil are considered to be generalists, and only a few of them are considered to be specialists, because soil-borne pathogens of grasses have a relative broad host range owing to the high phylogenetical relatedness of Poaceae (Barrett and Heil, 2012; Heinen *et al.*, 2020). Therefore, when heterospecific pathogens are generalists, they would have negative effects on the plant biomass of both *L. chinensis* and *C. squarrosa*; however, when heterospecific pathogens are specialists, they would not attack other plants in the absence of host plants (Semchenko *et al.*, 2022). Second, mycorrhizal symbiosis might not be strong enough to compensate for the loss of biomass caused by pathogens in our study site. We found that AMF functions were weak, because the mean root colonization rates were <15% with the non-sterilized treatment for both *L. chinensis* and *C. Squarrosa*. In fact, the AMF functions might generally not be as powerful on the Inner Mongolian grasslands as on the North American tallgrass prairies, owing to the low levels of root colonization (Jumpponen *et al.*, 2005; Johnson *et al.*, 2010; Tang *et al.*, 2020). Indeed, the AMF infections of *L. chinensis* and *C. squarrosa* were all <10% *in situ* (Guo *et al.*, 2016). Third, soil sterilization might lead to a small amount of nutrient release from dead microbial cell lysis, which could facilitate plant growth to a small degree. The results showed that sterilization would inevitably increase indigenous soil N and P concentrations. We adopted two ways to eliminate the nutrient leaching effects: (1) we used smaller amounts of indigenous soil for the inoculation to reduce the total amount of nutrient release; and (2) we used a stratified arrangement to reduce the differences in soil properties caused by sterilization (Adomako *et al.*, 2020). With regard to the N release, given that we created a N-rich environment, the excess mineral N released by sterilization might not theoretically increase plant growth (Bai *et al.*, 2010). Regarding the release of P, a similar increase in the available P concentration *in situ* would not significantly affect the *L. chinensis* and *C. squarrosa* biomass (Long *et al.*, 2016; Zhan *et al.*, 2017). Taken together, we attribute such effects of soil sterilization that cause plant biomass accumulation primarily to soil pathogens and to a lesser extent to nutrient release as a result of sterilization.

Putative pathogens are the primary drivers of negative plant–soil feedback (Ampt *et al.*, 2018; Semchenko *et al.*, 2018). The expression of defence genes can be used as an indicator when plants are in stressful conditions (Andersen *et al.*, 2018). There were 44 defence genes among the 164 annotated soil microbiota-specific genes, indicating that the plant defence system is activated by the soil microbiota. Signal transduction is the first response to occur after a pathogen is detected by a plant (Cui *et al.*, 2015). Plants use pathogen-associated molecular patterns rapidly to discern ‘non-self’ components, which include heat shock proteins and ubiquitin carbon (Tang *et al.*, 2019), and afterwards, the defence system is stimulated. Obliteration of reactive oxygen species is an essential process to protect cells from injury. Enzymes associated with obliteration of reactive oxygen species, such as superoxide dismutase, chalcone synthase and glutathione *S*-transferase, were identified in the present study (Noctor *et al.*, 2017; Rey *et al.*, 2019; Mittler *et al.*, 2022). The upregulation of glutathione *S*-transferase and superoxide dismutase also implies the degradation of exogenous toxins (Lee *et al.*, 2011). The most direct evidence comes from pathogenesis-related proteins and stress response upregulation, which indicates that the plant is under threat and that phytoalexin is accumulating (Conrath *et al.*, 2015; Noctor and Mhamdi, 2017). Furthermore, we found that the genes involved in oxidation and defence-encoding enzymes were upregulated by the soil microbiota. For example, chitinase is a major antifungal component that is used to degrade or break the pathogenic cell wall and inhibit fungal spore germination (Stressmann *et al.*, 2004).

### Phosphorus regulates soil microbial effects

From the perspective of biomass, we found that P addition could enhance the biomass of *L. chinensis* and *C. squarrosa*, which was consistent with the results of previous studies showing that increasing the nutrient supply could benefit plant growth and thus biomass accumulation (Ceulemans *et al.*, 2013). More importantly, we found that the addition of P could significantly alleviate the reduction in *C. squarrosa* biomass caused by the soil microbiota. Several possible mechanisms might explain how P regulates the soil microbial effects. First, P addition might boost the plant immune system and thus enable the plant to reduce the excessive levels of resources they need to allocate to their defence system (Shan *et al.*, 2018). This was supported by our findings that the expression of defence genes induced by soil microbiota started to downregulate after the P addition. Second, P addition might have reduced the intensity of resource competition between the plants and soil microbiota, being a limited and crucial nutrient for both plants and microbes (Zhou *et al.*, 2016). This was supported by the fact that the number of soil microbiota DEGs reduced tremendously after the P addition (from 7526 in low P to 2548 in high P) and that the expression levels of most upregulated soil microbiota-specific genes decreased after the P addition. For example, as a high-affinity inorganic phosphate transporter, the downregulation of PHO84 might imply that the P limitation is relieved (Jiang *et al.*, 2018). Third, the addition of P might change the soil microbiota community structure, which might switch the effects of the soil microbiota from negative to positive (Mezeli *et al.*, 2020).

From the perspective of the P concentration, we found that the presence of soil microbiota increased the shoot and total P concentrations of the *C. squarrosa* in the presence of low P. Furthermore, we found that soil sterilization significantly decreased soil alkaline phosphatase (AKP) activities and increased pH in the *C. squarrosa* monoculture. We could infer that the presence of the soil microbiota helped the *C. squarrosa* to obtain P by increasing the soil AKP activity and lowering the pH in the low P conditions. Owing to the extremely low AMF colonization rates for *C. squarrosa*, we attributed the function of P acquisition to soil P-solubilizing microorganisms. Soil P-solubilizing microorganisms can secrete organic acids or AKP to excavate more P to enable self-growth, especially in alkaline and low P soil conditions, hence plants could benefit from extra mobilized P (Jones and Oburger, 2011; Yao *et al.*, 2011). In contrast, we found that the soil sterilization treatment had no significant effects on the P concentration of *L. chinensis*. A recent study reports that *L. chinensis* has higher P resorption efficiency than *C. squarrosa* and that it relies more on internal nutrient cycling under high levels of grazing intensity (Wang *et al.*, 2020). In addition, root exudates of *L. chinensis* are rich in organic acids and amino acids, which could reduce pH and enhance P availability in rhizosphere soils (Lin *et al.*, 2022). This is evidenced by a field study demonstrating that the available P concentration in rhizosphere soils of *L. chinensis* is much higher than that of other perennial bunchgrasses in low P conditions (Zhan *et al.*, 2017).

### Planting patterns regulate soil microbial effects

We found that the planting pattern affected soil microbial effects significantly, with the mixture treatment alleviating the negative soil microbial effects on *C. squarrosa* but exaggerating those on *L. chinensis*. Three mechanisms could explain this contradictory pattern. First, common mycorrhizal networks might regulate the competitive relationship between *L. chinensis* and *C. squarrosa*. Muneer *et al.* (2023) reported that *C. squarrosa* showed greater potential for nutrient acquisition than *L. chinensis* in the dual common mycorrhizal network system, suggesting that *C. squarrosa* had a higher competitive ability than *L. chinensis* in the mixture. Second, certain soil pathogens that have severe negative impacts on *C. squarrosa* in the monoculture might then infect *L. chinensis* in the mixture. Based on the pathogen host specificity and negative density-dependent hypotheses (van Ruijven *et al.*, 2020), negative soil microbial effects will be alleviated if the neighbouring plant dilutes the pathogen abundance or exudes antifungal compounds (Rottstock *et al.*, 2014). It appears that *C. squarrosa* might harbour both generalist and specialist fungal pathogens in monoculture. The abundance of specialist fungal pathogens might be diluted, but the generalist fungal pathogens might then lead to more damage to the *L. chinensis* in the mixture. Third, the identity of neighbouring species might mediate the soil microbial effect and thus determine disease transmission (Yang *et al.*, 2014; Frank *et al.*, 2015). *L. chinensis* has well-developed rhizomes and higher root densities that could form root barricades to hamper soil-borne pathogen navigation. For *C. squarrosa*, the ‘root wall’ built by *L. chinensis* could block the spread of pathogens such that the negative soil microbial effects would be alleviated when it grows with *L. chinensis*. For *L. chinensis*, however, the root of *C. squarrosa* is not as strong as its own at obstructing pathogens; therefore, the negative soil microbial effects would be exaggerated when it grows with *C. squarrosa*.

### Conclusions

In this study, we established a glasshouse experiment to explore the effects of the indigenous soil microbiota on the growth of two dominant plant species, *L. chinensis* and *C. squarrosa*, in a semiarid grassland and examined how these effects were regulated by soil P availability and different planting patterns. Our results demonstrated that the presence of soil microbiota significantly decreased the total biomass of these two plant species in both monoculture and mixture conditions. Addition of P could reduce the negative microbial effects on both *L. chinensis* and *C. squarrosa*. The mixture treatment was found to alleviate the negative soil microbial effects on *C. squarrosa* biomass accumulation but to amplify the negative microbial effects on *L. chinensis*. These negative microbial effects could be attributed to the adverse effects of soil pathogens on plants, which induce the upregulation of a number of plant defence genes. However, addition of P could reduce the number of differentially expressed genes caused by soil microbiota and lead to the downregulation of some defence genes. These findings highlight that the varied outcomes of plant performance are affected by indigenous soil microbiota with different abiotic and biotic regulating factors. This study will help to enrich our molecular mechanistic understanding of the ecological functions of soil microbiota on plant communities in grasslands under the influences of climate change and anthropogenic activities.

## SUPPLEMENTARY DATA

Supplementary data are available online at https://academic.oup.com/aob and consist of the following. Table S1: results of homogeneity of variance and normality test for P concentration and microbial effects. Table S2: soil substrate nutrient analysis before and after γ-irradiation. Table S3: results of the three-way ANOVA for the effects of soil sterilization, P level and planting pattern and their interactions with P concentration. Table S4: results of the two-way ANOVA for the microbial effects of P level, planting pattern and their interactions on *Cleistogenes squarrosa* and *Leymus chinensis*. Table S5: results of the three-way ANOVA for the effects of soil sterilization, P level and planting pattern and their interactions with soil properties. Table S6: results of arbuscular mycorrhizal fungi (AMF) colonization rate. Table S7: soil microbiota-specific genes involved in defence processes. Table S8: soil microbiota-specific genes, excluding genes enriched in the ribosome pathway and defence genes. Fig. S1: results of plate tests on the sterilized and non-sterilized indigenous soil, sand, and vermiculite by Luria Broth (LB) medium, Potato Dextrose Agar (PDA) medium, and Gauze’s medium No.1. Fig. S2: effects of P addition and soil sterilization on the shoot and root biomass in monoculture and mixture of C. s*quarrosa* and L. *chinensis*. Fig. S3: effects of P addition and soil sterilization on the shoot and root P concentrations in monoculture and mixture of C. *squarrosa* and L. *chinensis*. Fig. S4: examples of various arbuscular mycorrhizal fungi colonization intensities.

mcad012_suppl_Supplementary_Material
