# Supplementary material for: Phosphorus availability and planting patterns regulate soil microbial effects on plant performance in a semiarid steppe
Source: Ann Bot. 2023 Jan 20;131(7):1081–95. doi: 10.1093/aob/mcad012 (PMC10457034; doi:10.1093/aob/mcad012)
Supplement: mcad012_suppl_Supplementary_Material [file mcad012_suppl_supplementary_material.docx]

**SUPPLEMENTARY DATA**

**Table S1** Results of homogeneity of variance (Levene’s test) and normality test (Shapiro-Wilk test) for P concentration (mg/kg) and microbial effects.

| Item |  | homogeneity of variance | | normality | |
| --- | --- | --- | --- | --- | --- |
|  |  | *F* | *p* | *W* | *p* |
| *C. squarrosa*  P concentration | shoot | 0.34 | 0.92 | 0.92 | 0.06 |
|  | root | 0.50 | 0.82 | 0.98 | 0.91 |
|  | total | 0.41 | 0.88 | 0.93 | 0.08 |
|  |  |  |  |  |  |
| *L. chinensis*  P concentration | shoot | 0.60 | 0.74 | 0.93 | 0.08 |
|  | root | 0.20 | 0.98 | 0.96 | 0.40 |
|  | total | 0.61 | 0.74 | 0.90 | 0.02 |
|  | total  *ln*-transformed | 0.63 | 0.73 | 0.93 | 0.12 |
|  |  |  |  |  |  |
| microbial effects | *C. squarrosa* | 1.17 | 0.34 | 0.95 | 0.18 |
|  | *L. chinensis* | 2.21 | 0.11 | 0.87 | 0.00 |
|  | *L. chinensis*  *sin*-transformed | 1.30 | 0.30 | 0.95 | 0.19 |

**Table S2** Soil substrate nutrient analysis before and after γ-irradiation*. Data are means ± standard error (SE; n = 5). AS, after sterilization; BS, before sterilization.

| Item |  | C (%) | N (%) | P (mg/kg) | Available P (mg/kg) | NH_4_-N  (mg/kg) | NO_3_-N (mg/kg) |
| --- | --- | --- | --- | --- | --- | --- | --- |
| indigenous soil | AS | 2.05±0.02a | 0.20±0.00a | 366.50±6.33a | 14.36±0.36a | 145.60±7.22a | 32.69±1.97a |
|  | BS | 1.80±0.01b | 0.18±0.00b | 318.40±5.91b | 3.11±0.21b | 12.78±0.61b | 45.24±1.76b |
|  |  |  |  |  |  |  |  |
| sand | AS | 0.07±0.01a | 0.01±0.00a | 45.78±1.62a | 5.09±0.38a | —— | —— |
|  | BS | 0.07±0.00a | 0.01±0.00a | 40.11±3.09a | 4.12±0.22a | —— | —— |
|  |  |  |  |  |  |  |  |
| vermiculite | AS | 0.05±0.01a | 0.00±0.00a | 102.40±8.77a | 3.69±0.16b | —— | —— |
|  | BS | 0.02±0.00b | 0.00±0.00a | 85.80±6.97a | 4.99±0.19a | —— | —— |

*, C(%) and N(%) of sand and vermiculite had already below the detection limit of the elemental analyzer (range of C: 0.35mg—21.30mg, range of N: 0.05mg—3.11mg, maximum sample weight is 30mg), the data are for information only.

**Table S3** Results (*F*-values) of the three-way analysis of variance (ANOVA) test for the effects of soil sterilization (S), P level (P), and planting pattern (T) and their interactions on P concentration (mg/kg). Significant differences are reported as *, *p* < 0.05; **, *p* < 0.01; ***, *p* < 0.001 and **†** for *p* < 0.01.

|  | *Cleistogenes squarrosa* | | | *Leymus chinensis* | | |
| --- | --- | --- | --- | --- | --- | --- |
| Source | Shoot P (mg/kg) | Root P (mg/kg) | Total P (mg/kg) | Shoot P (mg/kg) | Root P (mg/kg) | Total P (mg/kg) |
| S | 4.92* | 0.18 | 4.92* | 0.07 | 1.22 | 0.00 |
| P | 12.92** | 7.37* | 16.72*** | 0.04 | 3.49† | 0.00 |
| T | 0.59 | 0.10 | 0.34 | 3.76† | 0.00 | 3.22† |
| S × P | 4.56* | 3.57 | 6.39* | 1.82 | 2.58 | 1.96 |
| S × T | 0.01 | 1.30† | 0.00 | 1.05 | 0.02 | 1.25 |
| P × T | 0.01 | 1.96 | 0.01 | 0.21 | 0.09 | 0.17 |
| S × P × T | 0.97 | 0.82 | 1.04 | 0.03 | 2.22 | 0.32 |

**Table S4** Results of the two-way analysis of variance (ANOVA) test for the microbial effects of P level (P), planting pattern (T) and their interactions on *C. squarrosa* and *L. chinensis*. Significant differences are reported as **, *p* < 0.01; ***, *p* < 0.001 and **†** for *p* < 0.01.

| Species | P level | | Planting pattern | | P level × planting pattern | |
| --- | --- | --- | --- | --- | --- | --- |
|  | *F* | η² | *F* | η² | *F* | η² |
| ***C. squarrosa*** | 35.24*** | 0.59 | 9.99** | 0.29 | 2.73 | 0.10 |
| ***L. chinensis*** | 11.87** | 0.33 | 3.2**†** | 0.12 | 0.13 | 0.01 |

**Table S5** Results (*F*-values) of the three-way analysis of variance (ANOVA) test for the effects of soil sterilization (S), P level (P), and planting pattern (T) and their interactions on soil properties. Abbreviations: ACP, soil acid phosphatase; AKP, soil alkaline phosphatase. Significant differences are reported as *, *p* < 0.05; **, *p* < 0.01; and ***, *p* < 0.001.

| **Source** | **ACP** | **AKP** | **Available P** | **Total P** | **Total N** | **Total C** | **pH** |
| --- | --- | --- | --- | --- | --- | --- | --- |
| S | 0.07 | 2.92 | 8.10** | 2.14 | 3.45 | 0.60 | 15.80*** |
| P | 0.32 | 0.13 | 42.43*** | 3.15 | 0.12 | 5.57* | 13.82** |
| T | 35.36*** | 13.91*** | 1.10 | 0.24 | 4.84* | 3.90* | 6.69** |
| S × P | 7.08* | 7.18* | 0.62 | 5.08* | 0.37 | 2.93 | 3.56 |
| S × T | 4.79* | 14.21*** | 11.04*** | 0.12 | 4.76* | 3.83* | 3.77* |
| P × T | 0.86 | 10.82*** | 0.18 | 0.39 | 1.48 | 1.47 | 0.43 |
| S × P × T | 0.49 | 4.34* | 0.04 | 0.26 | 1.78 | 4.28* | 1.18 |

**Table S6** Results of arbuscular mycorrhizal fungi (AMF) colonization rate (%). Data are means ± standard error (SE; n = 3). Abbreviations: LP, low P; HP, high P; S, soil sterilization; and NS, soil non-sterilization.

| Species | P level | Soil treatment | monoculture | mixture |
| --- | --- | --- | --- | --- |
| *C. squarrosa* | LP | S | 0.00±0.00 | 0.00±0.00 |
|  |  | NS | 0.10±0.08 | 0.63±0.48 |
|  | HP | S | 0.00±0.00 | 0.00±0.00 |
|  |  | NS | 0.02±0.02 | 0.10±0.10 |
|  |  |  |  |  |
| *L. chinensis* | LP | S | 0.00±0.00 | 0.00±0.00 |
|  |  | NS | 7.68±3.13 | 13.97±13.32 |
|  | HP | S | 0.00±0.00 | 0.00±0.00 |
|  |  | NS | 6.80±6.40 | 2.17±1.28 |

**Table S7** Soil microbiota-specific genes involved in defence processes. Expression ratios are calculated by FPKM, and putative functions are annotated in KEGG or KOG. Abbreviations: LP, low P; HP, high P; S, soil sterilization; and NS, soil non-sterilization.

| Sequence ID | Putative function | Expression ratios | | | |
| --- | --- | --- | --- | --- | --- |
|  |  | LP S | HP S | LP NS | HP NS |
| Signal transduction | |  |  |  |  |
| Cluster-76343.244975 | heat shock 70kDa protein 1/8 | 0.00 | 0.00 | 2.96 | 1.02 |
| Cluster-76343.197869 | heat shock 70kDa protein 1/8 | 0.00 | 0.00 | 23.39 | 20.93 |
| Cluster-76343.202505 | heat shock transcription factor | 0.00 | 0.00 | 1.73 | 0.52 |
| Cluster-76343.70787 | heat shock protein 90kDa beta | 0.00 | 0.00 | 21.32 | 5.65 |
| Cluster-76343.8146 | elongation factor 1-alpha | 0.00 | 0.01 | 4.34 | 0.96 |
| Cluster-76343.11052 | elongation factor 1-alpha | 0.00 | 0.00 | 5.47 | 1.21 |
| Cluster-126726.0 | elongation factor 2 | 0.00 | 0.00 | 0.94 | 0.23 |
| Cluster-76343.243111 | elongation factor 3 | 0.00 | 0.00 | 3.61 | 1.04 |
| Cluster-76343.68759 | MADS-box transcription factor | 0.00 | 1.36 | 4.83 | 6.92 |
| Cluster-126317.0 | Ca^2+^ transporting ATPase | 0.00 | 0.00 | 1.02 | 0.38 |
| Cluster-76343.77441 | thioredoxin 1 | 5.35 | 5.11 | 0.51 | 0.20 |
| Cluster-76343.77435 | thioredoxin 1 | 55.99 | 51.09 | 1.49 | 1.38 |
| Cluster-76343.69567 | E3 ubiquitin-protein ligase | 58.83 | 55.24 | 9.87 | 12.05 |
| Cluster-76343.113562 | heterogeneous nuclear ribonucleoprotein G | 19.04 | 14.10 | 0.00 | 0.00 |
| Cluster-76343.10532 | Ubiquitin and ubiquitin-like proteins | 0.00 | 0.00 | 24.41 | 9.75 |
| Cluster-76343.83127 | Ubiquitin and ubiquitin-like proteins | 0.00 | 0.00 | 79.81 | 28.96 |
| Cluster-76343.6957 | EREBP-like factor | 1.07 | 0.63 | 5.37 | 4.87 |
| Cluster-76343.88501 | mitogen-activated protein kinase 6 | 0.37 | 0.20 | 0 | 0 |
| ROS & Pathogen-related protein | |  |  |  |  |
| Cluster-76343.190119 | pathogenesis-related protein 1 | 19.94 | 2.34 | 518.60 | 271.79 |
| Cluster-77846.0 | Stress responsive protein | 0.00 | 0.00 | 6.04 | 1.51 |
| Cluster-87332.0 | Stress responsive protein | 0.00 | 0.00 | 32.15 | 6.78 |
| Cluster-76343.116758 | disease resistance protein RPM1 | 8.26 | 6.40 | 0.00 | 0.00 |
| Cluster-76343.58707 | glutathione S-transferase | 0.03 | 0.00 | 60.22 | 28.21 |
| Cluster-76343.51200 | serine carboxypeptidase-like clade II | 0.05 | 0.03 | 23.16 | 11.52 |
| Cluster-76343.150035 | chalcone synthase | 0.34 | 0.08 | 30.02 | 11.10 |
| Cluster-76343.141057 | cysteine and glycine-rich protein | 13.68 | 5.96 | 0.00 | 0.00 |
| Other Enzymes |  |  |  |  |  |
| Cluster-76343.196737 | KDEL-tailed cysteine endopeptidase | 0.00 | 0.00 | 6.95 | 2.16 |
| Cluster-76343.58190 | KDEL-tailed cysteine endopeptidase | 0.00 | 0.00 | 145.01 | 44.46 |
| Cluster-76343.41591 | KDEL-tailed cysteine endopeptidase | 0.02 | 0.02 | 45.31 | 10.89 |
| Cluster-76343.71912 | KDEL-tailed cysteine endopeptidase | 0.00 | 0.04 | 116.46 | 33.74 |
| Cluster-76343.214568 | 6,7-dimethyl-8-ribityllumazine synthase | 0.00 | 0.00 | 11.57 | 5.13 |
| Cluster-76343.20658 | 6,7-dimethyl-8-ribityllumazine synthase | 0.00 | 0.00 | 7.68 | 2.53 |
| Cluster-76343.249754 | chitinase | 0.09 | 0.06 | 23.46 | 8.35 |
| Cluster-82242.0 | chitinase | 0.12 | 0.22 | 1.86 | 2.57 |
| Cluster-76343.192504 | catalase | 0.00 | 0.01 | 7.20 | 2.41 |
| Cluster-76343.10135 | superoxide dismutase, Fe-Mn family | 0.00 | 0.00 | 11.54 | 3.50 |
| Cluster-76343.47682 | superoxide dismutase, Fe-Mn family | 0.00 | 0.00 | 7.79 | 2.00 |
| Cluster-78864.0 | Manganese superoxide dismutase | 0.00 | 0.00 | 1.88 | 0.58 |
| Cluster-76343.71242 | caffeic acid 3-O-methyltransferase | 0.14 | 0.04 | 9.01 | 6.56 |
| Cluster-76343.71247 | caffeic acid 3-O-methyltransferase | 1.04 | 0.41 | 51.75 | 43.78 |
| Oxidation |  |  |  |  |  |
| Cluster-116222.2 | H^+^-transporting ATPase | 0.00 | 0.00 | 0.89 | 0.41 |
| Cluster-76343.165091 | F-type H^+^-transporting ATPase subunit O | 0.00 | 0.00 | 1.53 | 1.96 |
| Cluster-76343.186977 | Oxidation resistance protein | 8.60 | 7.03 | 0.00 | 0.00 |
| Cluster-77434.0 | inorganic pyrophosphatase | 0.00 | 0.00 | 1.65 | 0.56 |

**Table S8** Soil microbiota-specific genes which excluded genes enriched in ribosome pathway and defense genes. Expression ratios are calculated by FPKM. Abbreviations: LP, low P; HP, high P; S, soil sterilization; and NS, soil non-sterilization.

| ID | LP S | HP S | LP NS | HP NS | KO Name | KO Description | KOG Description |
| --- | --- | --- | --- | --- | --- | --- | --- |
| Cluster-109823.0 | 0.00 | 0.00 | 6.89 | 2.35 | EGD1, BTF3 | nascent polypeptide-associated complex subunit beta | RNA polymerase II general transcription factor BTF3 and related proteins |
| Cluster-113225.0 | 0.00 | 0.00 | 2.74 | 1.08 | THI4, THI1 | thiamine thiazole synthase | Protein involved in thiamine biosynthesis and DNA damage tolerance |
| Cluster-121269.0 | 0.00 | 0.00 | 2.18 | 0.88 | -- | -- | Predicted short chain-type dehydrogenase |
| Cluster-124281.0 | 0.00 | 0.00 | 4.40 | 1.23 | MARS, metG | methionyl-tRNA synthetase | tRNA-binding protein |
| Cluster-126113.0 | 0.00 | 0.00 | 1.18 | 0.23 | -- | -- | Maltase glucoamylase and related hydrolases, glycosyl hydrolase family 31 |
| Cluster-128350.0 | 0.00 | 0.00 | 4.33 | 2.00 | SSADH | succinate-semialdehyde dehydrogenase, mitochondrial | Aldehyde dehydrogenase |
| Cluster-129369.2 | 0.00 | 0.00 | 0.46 | 0.51 | ABCF3 | ATP-binding cassette, subfamily F, member 3 | ATPase component of ABC transporters with duplicated ATPase domains/Translation elongation factor EF-3b |
| Cluster-129382.0 | 0.00 | 0.00 | 1.37 | 0.37 | NBR1 | next to BRCA1 gene 1 protein | Uncharacterized conserved protein, contains ZZ-type Zn-finger |
| Cluster-76343.104720 | 13.77 | 8.93 | 0.00 | 0.00 | -- | -- | Nuclear transport factor 2 |
| Cluster-76343.106094 | 24.38 | 8.68 | 0.00 | 0.00 | SLU7 | pre-mRNA-processing factor SLU7 | RNA splicing factor - Slu7p |
| Cluster-76343.110218 | 45.26 | 14.91 | 0.05 | 0.00 | SLC35D | solute carrier family 35 | -- |
| Cluster-76343.111953 | 2.56 | 1.42 | 0.00 | 0.00 | MAK21, NOC1, CEBPZ | ribosome biogenesis protein MAK21 | CAATT-binding transcription factor/60S ribosomal subunit biogenesis protein |
| Cluster-76343.11754 | 0.01 | 0.00 | 11.43 | 3.64 | PGD, gnd, gntZ | 6-phosphogluconate dehydrogenase | 6-phosphogluconate dehydrogenase |
| Cluster-76343.119865 | 4.18 | 2.36 | 0.11 | 0.00 | -- | -- | Sodium sulfate symporter and related arsenite permeases |
| Cluster-76343.129406 | 8.73 | 4.57 | 0.02 | 0.02 | PCYT2 | ethanolamine-phosphate cytidylyltransferase | Choline phosphate cytidylyltransferase/Predicted CDP-ethanolamine synthase |
| Cluster-76343.129482 | 3.16 | 3.21 | 0.11 | 0.10 | SEC24 | protein transport protein SEC24 | Vesicle coat complex COPII, subunit SEC24/subunit SFB2 |
| Cluster-76343.132213 | 37.32 | 27.55 | 0.00 | 0.07 | ALDO | fructose-bisphosphate aldolase, class I | Fructose-biphosphate aldolase |
| Cluster-76343.133257 | 26.01 | 16.34 | 0.24 | 0.16 | SMARCAL1, HARP | SWI/SNF-related matrix-associated actin-dependent regulator of chromatin subfamily A-like protein 1 | Chromatin remodeling protein HARP/SMARCAL1, DEAD-box superfamily |
| Cluster-76343.13527 | 0.04 | 0.00 | 7.49 | 2.53 | HGSNAT | heparan-alpha-glucosaminide N-acetyltransferase | Uncharacterized conserved protein |
| Cluster-76343.13551 | 0.00 | 0.00 | 7.14 | 1.93 | -- | -- | WD40 repeat-containing protein |
| Cluster-76343.140635 | 3.46 | 2.39 | 0.18 | 0.14 | MAK16 | protein MAK16 | RNA-binding nuclear protein (MAK16) containing a distinct C4 Zn-finger |
| Cluster-76343.146569 | 0.04 | 0.04 | 15.48 | 8.77 | PHO84 | MFS transporter, PHS family, inorganic phosphate transporter | -- |
| Cluster-76343.154146 | 48.35 | 36.45 | 0.15 | 0.25 | SFRS7 | splicing factor, arginine/serine-rich 7 | Alternative splicing factor SRp20/9G8 (RRM superfamily) |
| Cluster-76343.161989 | 2.66 | 0.09 | 0.12 | 1.73 | -- | -- | Ankyrin |
| Cluster-76343.166518 | 13.21 | 11.10 | 0.00 | 0.00 | CTSF | cathepsin F | Cysteine proteinase Cathepsin F |
| Cluster-76343.166519 | 13.13 | 10.63 | 0.00 | 0.00 | CTSF | cathepsin F | Cysteine proteinase Cathepsin F |
| Cluster-76343.186234 | 15.03 | 7.51 | 0.24 | 0.27 | aguA | agmatine deiminase | -- |
| Cluster-76343.189153 | 0.00 | 0.00 | 69.77 | 19.98 | TUBA | tubulin alpha | Alpha tubulin |
| Cluster-76343.189155 | 0.00 | 0.00 | 22.97 | 10.10 | TUBA | tubulin alpha | Alpha tubulin |
| Cluster-76343.192026 | 0.00 | 0.00 | 14.91 | 3.76 | -- | -- | Karyopherin (importin) alpha |
| Cluster-76343.19606 | 0.00 | 0.00 | 4.04 | 0.97 | PDIA1, P4HB | protein disulfide-isomerase A1 | Protein disulfide isomerase (prolyl 4-hydroxylase beta subunit) |
| Cluster-76343.19615 | 0.00 | 0.00 | 3.69 | 1.48 | SSRP1 | structure-specific recognition protein 1 | HMG box-containing protein |
| Cluster-76343.197868 | 0.00 | 0.00 | 27.43 | 9.59 | HSPA1_8 | heat shock 70kDa protein 1/8 | Molecular chaperones HSP70/HSC70, HSP70 superfamily |
| Cluster-76343.19993 | 0.00 | 0.00 | 7.78 | 1.90 | -- | -- | Alcohol dehydrogenase, class III |
| Cluster-76343.203444 | 0.00 | 0.00 | 1.92 | 0.71 | -- | -- | Vacuolar protein sorting-associated protein |
| Cluster-76343.204868 | 1.74 | 1.35 | 0.00 | 0.00 | -- | -- | Amidases |
| Cluster-76343.20674 | 0.00 | 0.00 | 13.79 | 3.83 | -- | -- | Uncharacterized conserved protein |
| Cluster-76343.209653 | 0.00 | 0.00 | 2.72 | 0.57 | -- | -- | Fibrillins and related proteins containing Ca2+-binding EGF-like domains |
| Cluster-76343.210702 | 0.00 | 0.00 | 5.09 | 1.96 | -- | -- | Predicted Zn-finger protein |
| Cluster-76343.212339 | 0.00 | 0.00 | 5.04 | 1.52 | E2.1.1.77, pcm | protein-L-isoaspartate(D-aspartate) O-methyltransferase | Protein-L-isoaspartate(D-aspartate) O-methyltransferase |
| Cluster-76343.213678 | 0.00 | 0.00 | 15.44 | 4.76 | -- | -- | Sorbitol dehydrogenase |
| Cluster-76343.214130 | 0.02 | 0.02 | 8.84 | 8.24 | amt, AMT, MEP | ammonium transporter, Amt family | Ammonia permease |
| Cluster-76343.214132 | 0.00 | 0.02 | 6.33 | 4.18 | amt, AMT, MEP | ammonium transporter, Amt family | Ammonia permease |
| Cluster-76343.218734 | 0.00 | 0.00 | 20.19 | 5.35 | ACTF | actin, other eukaryote | Actin and related proteins |
| Cluster-76343.22678 | 0.00 | 0.99 | 0.75 | 0.00 | mutM, fpg | formamidopyrimidine-DNA glycosylase | -- |
| Cluster-76343.231471 | 7.05 | 5.53 | 0.07 | 0.00 | COPS8, CSN8 | COP9 signalosome complex subunit 8 | -- |
| Cluster-76343.243415 | 0.00 | 0.00 | 5.98 | 1.44 | -- | -- | Uncharacterized conserved protein |
| Cluster-76343.244855 | 0.00 | 0.00 | 1.63 | 0.85 | PRPF4B | serine/threonine-protein kinase PRP4 | U4/U6-associated splicing factor PRP4 |
| Cluster-76343.247649 | 0.00 | 0.00 | 4.27 | 1.52 | DIS3, RRP44 | exosome complex exonuclease DIS3/RRP44 | Exosomal 3'-5' exoribonuclease complex, subunit Rrp44/Dis3 |
| Cluster-76343.248117 | 0.00 | 0.00 | 5.84 | 1.57 | -- | -- | Cytochrome oxidase assembly factor COX15 |
| Cluster-76343.248439 | 0.00 | 0.00 | 2.14 | 1.09 | ACSL, fadD | long-chain acyl-CoA synthetase | Acyl-CoA synthetase |
| Cluster-76343.2927 | 5.14 | 0.63 | 0.14 | 0.00 | -- | -- | Predicted lipase/calmodulin-binding heat-shock protein |
| Cluster-76343.35858 | 26.97 | 11.49 | 5.49 | 1.73 | SLC50A, SWEET | solute carrier family 50 (sugar transporter) | -- |
| Cluster-76343.49623 | 0.13 | 0.01 | 17.91 | 4.75 | -- | -- | Acyl-CoA synthetase |
| Cluster-76343.51956 | 5.74 | 5.96 | 1.89 | 1.71 | MARS, metG | methionyl-tRNA synthetase | tRNA-binding protein |
| Cluster-76343.54466 | 0.00 | 0.00 | 8.60 | 2.40 | -- | -- | Septin family protein (P-loop GTPase) |
| Cluster-76343.60649 | 6.76 | 1.95 | 0.00 | 0.00 | -- | -- | Acyl-CoA synthetase |
| Cluster-76343.64012 | 0.00 | 0.00 | 6.66 | 2.04 | ACTF | actin, other eukaryote | Actin and related proteins |
| Cluster-76343.72515 | 0.00 | 0.00 | 52.13 | 6.14 | H2B | histone H2B | Histone H2B |
| Cluster-76343.73163 | 0.03 | 0.00 | 57.82 | 26.11 | PHO84 | MFS transporter, PHS family, inorganic phosphate transporter | -- |
| Cluster-76343.79454 | 15.43 | 9.17 | 0.05 | 0.10 | E1.2.1.3 | aldehyde dehydrogenase (NAD+) | Aldehyde dehydrogenase |
| Cluster-76343.94842 | 0.00 | 0.00 | 13.25 | 4.21 | YWHAE | 14-3-3 protein epsilon | Multifunctional chaperone (14-3-3 family) |
| Cluster-76343.95325 | 0.00 | 0.00 | 0.57 | 0.54 | -- | -- | Transcription factor CtBP |
| Cluster-76761.0 | 0.00 | 0.00 | 0.91 | 0.69 | -- | -- | Predicted membrane protein |
| Cluster-77846.0 | 0.00 | 0.00 | 6.04 | 1.51 | -- | -- | Stress responsive protein |
| Cluster-78795.0 | 0.00 | 0.00 | 4.61 | 2.30 | -- | -- | Alcohol dehydrogenase, class III |
| Cluster-84336.0 | 0.00 | 0.00 | 1.01 | 0.29 | ACSBG | long-chain-fatty-acid--CoA ligase ACSBG | Long-chain acyl-CoA synthetases (AMP-forming) |
| Cluster-92914.0 | 0.00 | 0.00 | 4.77 | 1.47 | YWHAE | 14-3-3 protein epsilon | Multifunctional chaperone (14-3-3 family) |

**
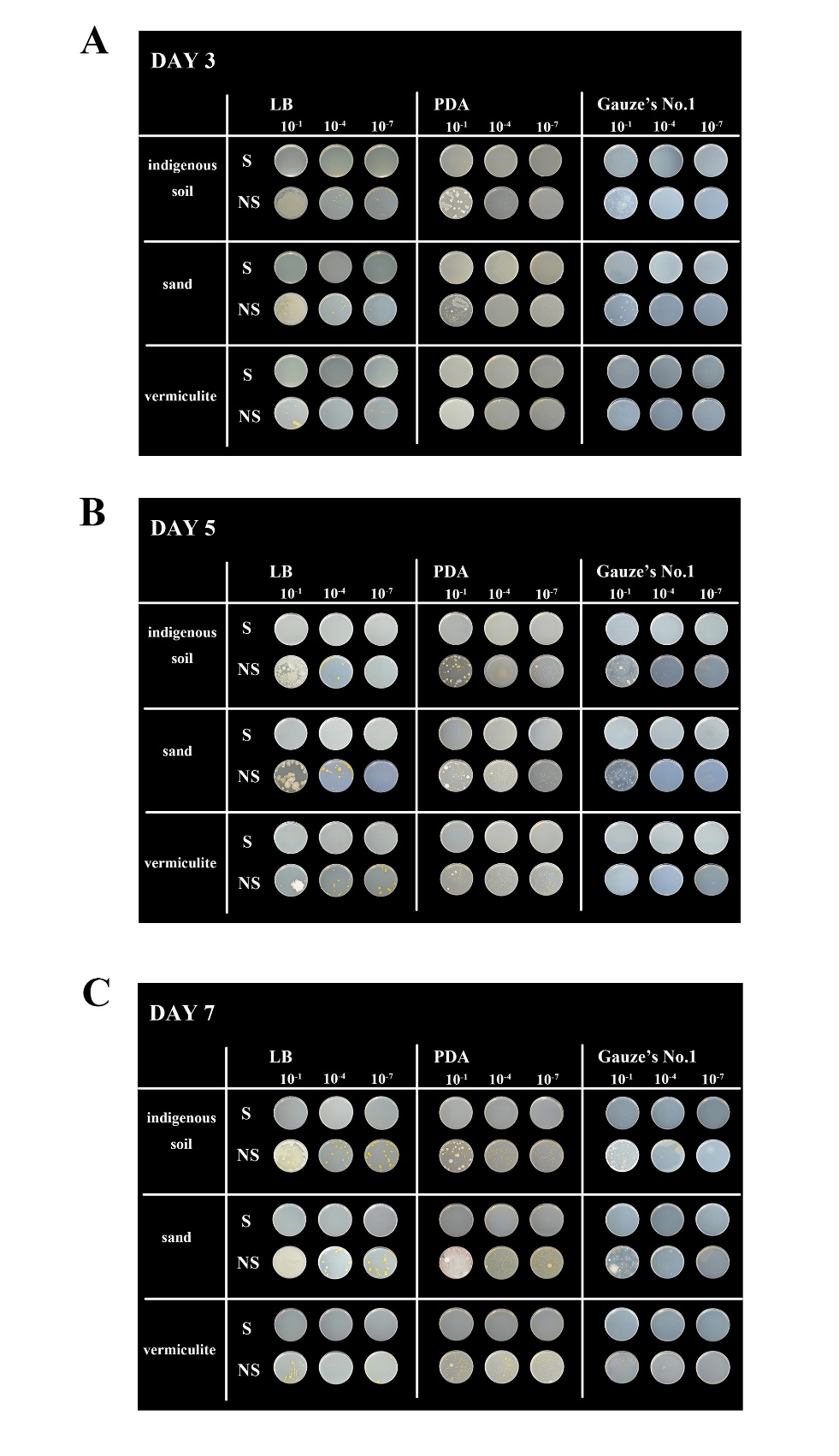
**

**Figure S1.** Results of plate tests on the sterilized and non-sterilized indigenous soil, sand, and vermiculite by Luria Broth (LB) medium, Potato Dextrose Agar (PDA) medium, and Gauze’s medium No.1. Results from day3, day5, and day7 with dilution gradients of 10^-1^, 10^-4^, 10^-7^ were presented in (A), (B), and (C), respectively. **Abbreviations:** S, soil sterilization; NS, soil non-sterilization.

**
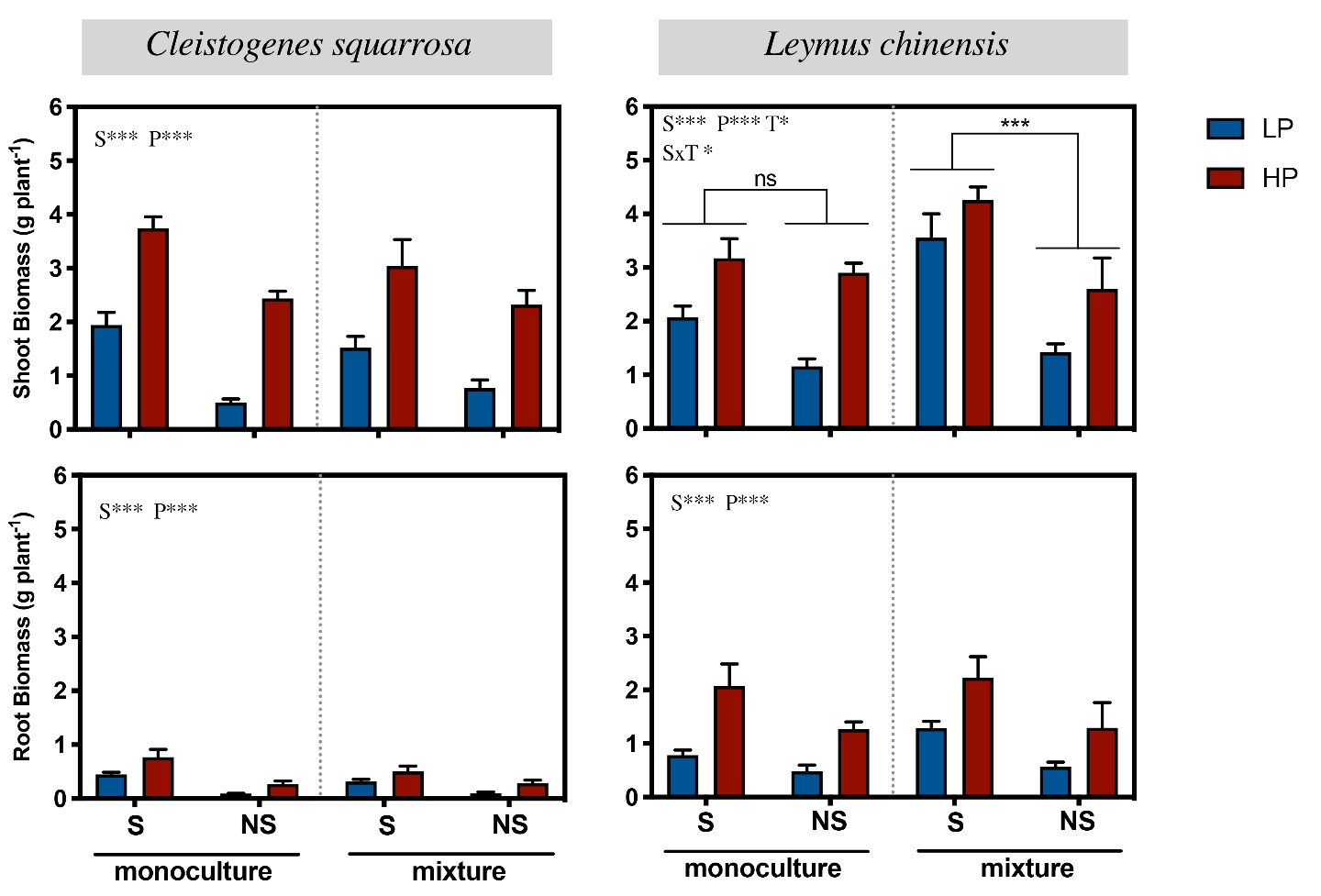
**

**Figure S2.** Effects of P addition and soil sterilization on the shoot and root biomass in monoculture and mixture of *Cleistogenes squarrosa* and *Leymus chinensis*. Bars represent means ± standard error (SE; n = 7). **p* < 0.05, ****p* < 0.001. ns represents no significant effects at *p* < 0.05. **Abbreviations:** LP, low P; HP, high P; S, soil sterilization; NS, soil non-sterilization. P, P level; T, planting pattern.

**
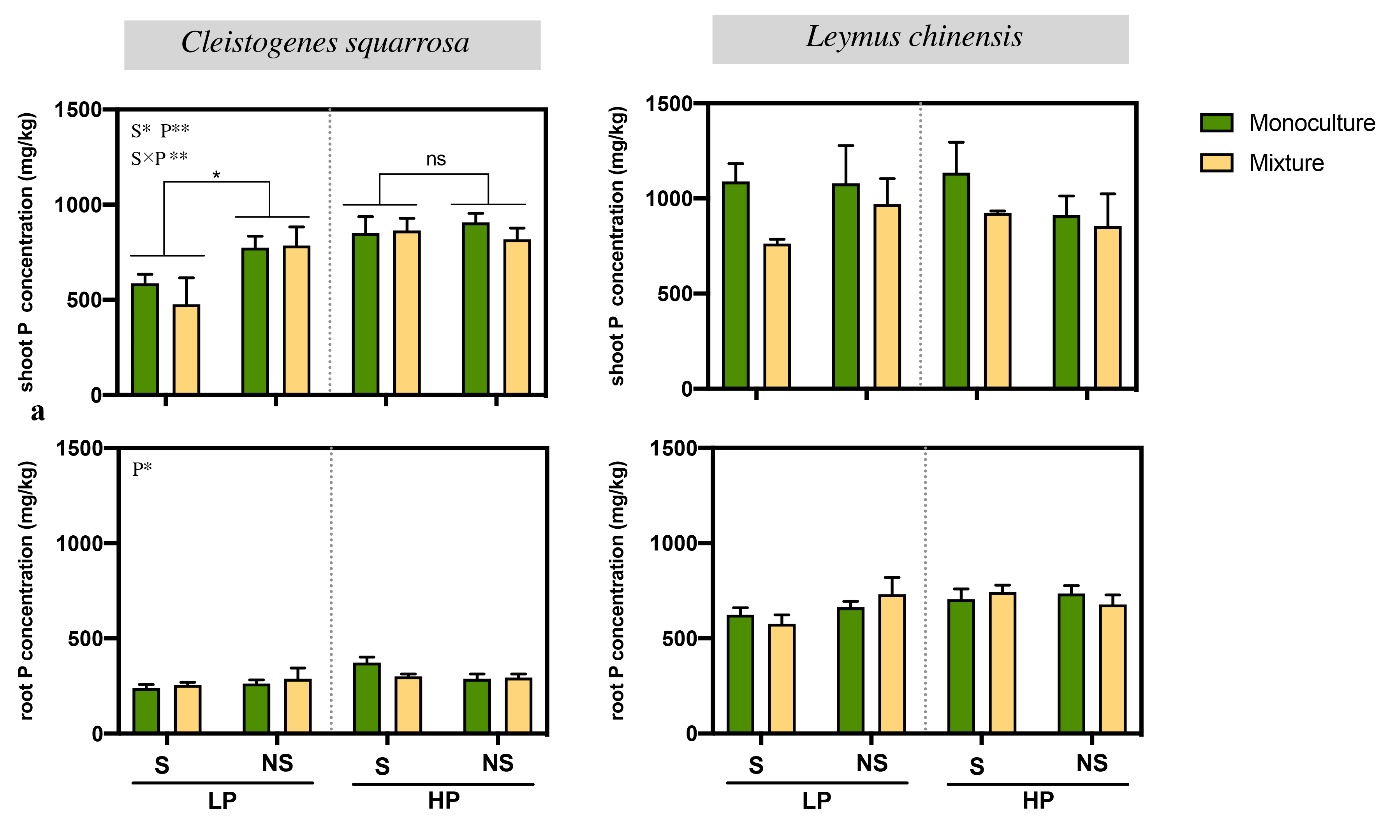
**

**Figure S3.** Effects of P addition and soil sterilization on the shoot and root P concentrations (mg/kg) in monoculture and mixture of *Cleistogenes squarrosa* and *Leymus chinensis*. Bars represent means ± standard error (SE; n = 3). **Abbreviations:** LP, low P; HP, high P; S, soil sterilization; NS, soil non-sterilization. **p* < 0.05, ****p* < 0.001. ns represents no significant effects at *p* < 0.05. Insignificant groups are not identified.

**
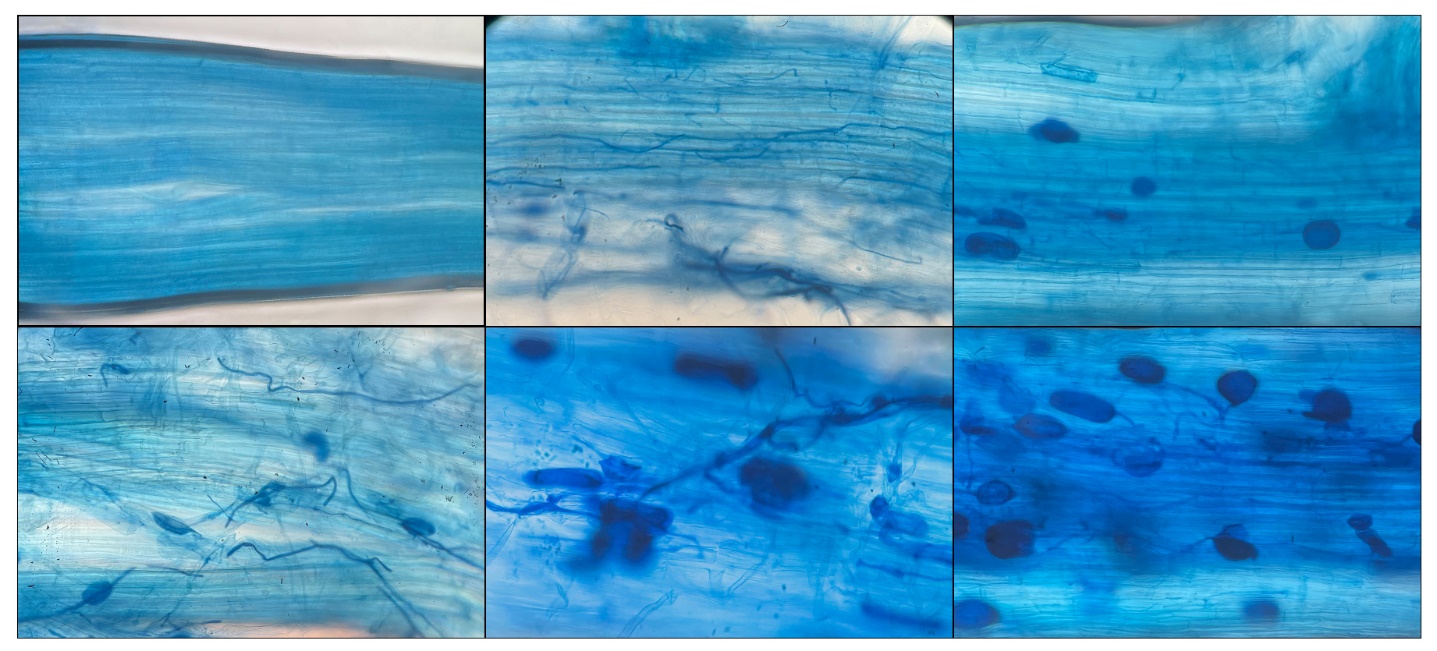
**

**Figure S4.** Examples of various arbuscular mycorrhizal fungi colonization intensities.
